# Validating Field Methods to Estimate the Pelvic Tilt in Sprinting and the Relationship between Prior Hamstring Injury and the Pelvic Tilt in Elite Female Soccer Players

**DOI:** 10.5114/jhk/194851

**Published:** 2025-06-25

**Authors:** András Hegyi, Aurélie Sarcher, Fabien Varenne, Alexis Mornet, Jean-Philippe Cadu, Lena Carcreff, Lilian Lacourpaille

**Affiliations:** 1 Department of Kinesiology, Hungarian University of Sports Science, Budapest, Hungary.; 2 Movement—Interactions—Performance, MIP, UR 4334, Nantes Université, Nantes, France.; 3 Performance Department, Football Club of Nantes, Nantes, France.

**Keywords:** biceps femoris, strain, kinematics, front-side mechanics, kick-back mechanism, lumbo-pelvic control

## Abstract

An excessive pelvic tilt in the late swing phase of sprinting may be associated with an increased risk of hamstring injury. Nevertheless, research including female athletes is scarce. Furthermore, it is essential to validate simple on-field methods. This study consisted of two experiments. Experiment I assessed the validity of two 2-D video-based methods: i) the kick-back score calculated from thigh angles at the toe-off and the touchdown; and ii) the pelvic tilt estimated by a line connecting two markers on the pelvis. Twelve soccer players sprinted for 30 m, and 3-D motion capture data and 2-D sagittal plane video were recorded. Experiment II aimed to compare the above 2-D methods in recently injured (n = 7) and non-injured (n = 18) professional female soccer players. In Experiment I, no correlation was found between the kick-back score and the pelvic tilt assessed using 3-D motion capture (rho = −0.224, p = 0.242). Two-D camera-based estimation of the pelvic tilt correlated with the 3-D pelvic tilt (r = 0.89–0.94, *p* < 0.001). In Experiment II, the kick-back score was not significantly different between groups (d = 0.11, p = 0.41). The pelvic tilt was higher in the previously injured than in non-injured players in the late swing phase (d = −0.79, p = 0.03). Our results suggest that the kick-back score is not associated with the pelvic tilt. Nevertheless, the estimation of the pelvic tilt in field settings is feasible through the tracking of two markers on the pelvis in the sagittal plane. Additionally, longitudinal studies are recommended to gain deeper understanding of the excessive pelvic tilt in previously injured female soccer players

## Introduction

Hamstring muscle strain injuries represent a significant challenge in sports that involve sprinting. These injuries predominantly manifest in the biceps femoris muscle during the late swing phase of sprinting*. In vitro* studies have demonstrated that increased muscle fibre strain and decreased ability to absorb energy within the muscle result in muscle failure (Garrett et al., 1987; Lieber and Fridén, 1993). Although quantifying muscle fibre strain *in vivo* remains a significant challenge, a number of intrinsic risk factors, which may affect fibre strain, have been identified. These include reduced maximal eccentric knee flexion strength (Bourne et al., 2015), short biceps femoris fascicle length (Timmins et al., 2016), high hamstring muscle-tendon unit stiffness (Watsford et al., 2010), altered intermuscular activation patterns (Schuermans et al., 2017a), and altered kinematics in the swing phase of sprinting (Kenneally-Dabrowski et al., 2019; Schuermans et al., 2017b). Regarding kinematics, the study of Schuermans et al. (2017b) suggests that an excessive anterior pelvic tilt in the late swing increases the risk of strain injury in the biceps femoris of male soccer players. This can be attributed to the fact that the biceps femoris muscle is originated from the ischial tuberosity (posterior-distal side of the pelvis). The pelvic tilt shows a biphasic pattern throughout the sprint cycle, with the highest anterior tilt occurring in the early swing (short hamstring length) and in the late swing (long hamstring length) (Higashihara et al., 2015; Nagano et al., 2014). In the late swing phase, which is when hamstring injuries most commonly occur, musculoskeletal modelling has shown that an increased anterior pelvic tilt leads to an increased strain in the biceps femoris (Thelen et al., 2006). However, it is important to note, that the pelvic tilt is typically measured relative to the horizontal plane, which does not take into account the position of the femur and tibia. Consequently, the hip and knee angles, which are crossed by the hamstrings, are not accurately represented. Furthermore, modelling studies do not take into account any compensatory mechanisms that may occur in real-life scenarios. Thus, further experimental evidence is required to elucidate the relationship between the pelvic tilt angle and hamstring length. Assuming that the pelvic tilt and the hamstring length are related, monitoring the pelvic tilt for the purposes of screening and assessing changes in response to training interventions is a valuable tool in many sports. The implementation of straightforward and validated methodologies is of paramount importance for the accurate assessment of the pelvic tilt in field settings.

To estimate pelvic tilt-related hamstring length deficits, Lahti et al. (2020) introduced the measurement of the kick-back score in male professional soccer players. The kick-back score is a composite score of two angles: the thigh angle (relative to the horizontal) at the toe-off of the same leg and the thigh angle at the foot strike of the contralateral leg. A higher kick-back score indicates a shift towards a front-side dominant technique, which is considered to be more optimal than a lower score, which indicates a more back-side dominant technique (Lahti et al., 2020). However, the kick-back score does not offer direct insight into the position of the pelvis. The concept of using thigh angle measurements to ascertain the pelvic tilt was derived from the observation that alterations in the pelvic tilt in response to training interventions were accompanied by corresponding changes in the thigh angle (Mendiguchia et al., 2022). Most studies that observed the pelvic tilt used three-dimensional (3-D) motion capture systems, while the kick-back score can be calculated from sagittal-plane two-dimensional (2-D) images. The kick-back score has the potential to become a popular measure in sprint-based sports due to its simplicity and the ease with which it can be calculated. Nevertheless, the relationship between the thigh angle relative to the horizontal plane and the incidence of hamstring injuries has not been previously established. Furthermore, the use of the kick-back score as a surrogate for the pelvic tilt remains to be validated. In lieu of using a sophisticated 3-D motion capture system, sagittal plane video analysis of reflective markers positioned on the pelvis may offer an estimation of the pelvic tilt angle with an acceptable degree of precision.

It is important to note that research relating the pelvic tilt to the incidence of hamstring injuries is limited. A previous study has shown that an excessive anterior pelvic tilt in sprinting may be associated with an elevated risk of hamstring injuries among soccer players (Schuermans et al., 2017b). Some retrospective studies on athletes engaged in sprint-based sports have identified differences in the pelvic tilt angle between those who have sustained injuries and those who have not (Daly et al., 2016; Higashihara et al., 2019). On the contrary, Kenneally-Dabrowski et al. (2019) did not identify the pelvic tilt as a risk factor for hamstring injuries in elite rugby players. Furthermore, the aforementioned studies exclusively focused on male athletes. Although the incidence of hamstring injuries is comparable or higher in male than in female soccer players, hamstring strain remains the most common injury type among female soccer players (Horan et al., 2022; Nilstad et al., 2014). From an anatomical standpoint, the female pelvis is distinguished by its relatively larger size, wider breadth, and the greater distance between the ischial tuberosities, which are also more medially projected (Fischer and Mitteroecker, 2017). It can be assumed that these anatomical features may amplify the impact of the pelvic tilt angle on the lengths of the hamstrings in female athletes. In contrast, the hamstrings of female athletes are more compliant (Blackburn et al., 2009), which may decrease the risk imposed by an anteriorly tilted pelvis. Therefore, studies are essential to elucidate the potential relationship between the pelvic tilt and the risk of hamstring injuries in female athletes.

To enhance our comprehension of the topic, a two-part experiment was designed. The aim of *Experiment I* was to examine the association between 1) the thigh angle (2-D video) and the pelvic tilt angle (3-D motion capture) at the toe-off and the foot strike; 2) the pelvic tilt angle (2-D video) and the pelvic tilt measured by a 3-D motion capture at the toe-off, the foot strike, and the late swing phase; and 3) the kick-back score (2-D video) and an equivalent composite score for the pelvic tilt (3-D motion capture). We also aimed to assess whether there was a correlation between the pelvic tilt and the length of the biceps femoris muscle-tendon unit in the late swing phase of sprinting. We hypothesized that, of the two video-based methods, estimation of the pelvic tilt based on 2-D video would show a positive correlation with the pelvic tilt as measured by 3-D motion capture. No correlation was expected between the pelvic tilt and the kick-back score. We also hypothesized that an increased anterior pelvic tilt would be correlated with longer biceps femoris length in the late swing phase of sprinting.

The objective of *Experiment II* was to investigate the differences in the pelvic tilt and the kick-back score between previously injured and non-injured soccer players. It was anticipated that the recently injured players would demonstrate a more pronounced anterior pelvic tilt and that there would be no significant difference in the kick-back score between the recently injured and non-injured players.

## Methods

### **Participants**

In both experiments, the maximum number of participants was limited by the constraints of available resources. Furthermore, there is a paucity of knowledge regarding the smallest effect sizes that are practically meaningful. This has hindered our ability to perform effective *a priori* power analyses.

In *Experiment I*, 12 healthy soccer players without previous hamstring injuries (7 females, 5 males, age 20 ± 3 years, body height 1.72 ± 0.1 m, body mass 67 ± 9 kg) were recruited from local soccer clubs. They had mean weekly practice duration of 9 ± 3 h. Further exclusion criteria were any lower-limb injury or surgery in the previous twelve months. In *Experiment II*, 25 professional female soccer players of FC Nantes participated (age 23 ± 4 years, body height 1.64 ± 0.04 m, body mass 58 ± 6 kg), seven of which had a hamstring injury 71 ± 32 days prior to the experiment (three injuries in the right leg). The remaining participants had no history of hamstring injury within the six months preceding the testing period. We aimed to include players from the same team in this experiment, thereby ensuring that both the injured and the non-injured were sampled from the same population. A hamstring injury was defined as an acute sudden onset of pain in the posterior thigh that occurred during training or match play resulting in immediate termination of activity and inability to participate in the subsequent training session or match. In all cases, the diagnosis was corroborated by a qualified medical practitioner. All participants provided informed written consent prior to participation, in accordance with the guidelines set by the Declaration of Helsinki. All procedures were approved by the Comité de protection des personnes Ile de France I (approval code: CPP IDF I, n°2018-A02675-50; approval date: 9 November 2021).

### **Measures**

In both experiments, 2-D kinematics were recorded using a high-resolution video camera (12MPx camera of iPhone XS, Apple Inc., CA, USA) placed 1.2 m high, at 15 m along the 30 m sprint distance, 10 m away from the running track, at a sampling frequency of 240 Hz. *Experiment I* was conducted within a stadium setting, necessitating the use of a strobe light to enhance the visibility of the retro-reflective markers on the 2-D video recordings. In *Experiment I*, 3-D kinematics were recorded using an 8-camera 3-D infrared motion analysis system (Vero 2.2, VICON, Oxford, UK) in the same space where 2-D data were collected, at a sampling frequency of 240 Hz. Players who had sustained an injury to their left leg were instructed to sprint in the opposite direction to the rest of the group to facilitate the collection of data from the injured side. The right leg was measured for all athletes without a recent hamstring injury due to the practical considerations of convenience and the absence of known inter-limb differences in the pelvic tilt in non-injured individuals.

### **Design and Procedures**

In both experiments, participants were instructed to wear black, tight-fitting clothing, specifically sports leggings. The black color of the clothing facilitated the identification of the retro- reflective markers on the 2-D video recordings during the analysis. Each participant completed the following standardized warm-up protocol: two 200-m running laps, followed by active dynamic stretching of the major leg muscle groups; then body-weight-only exercises including squats, single-leg hamstring bridges, lunges, and running drills (heel to butt, high knees, straight leg). Subsequently, participants engaged in 20–30-m sprints with gradually increasing intensity (target: 70%, 85%, and 100% of the maximum speed).

**Figure 1:**
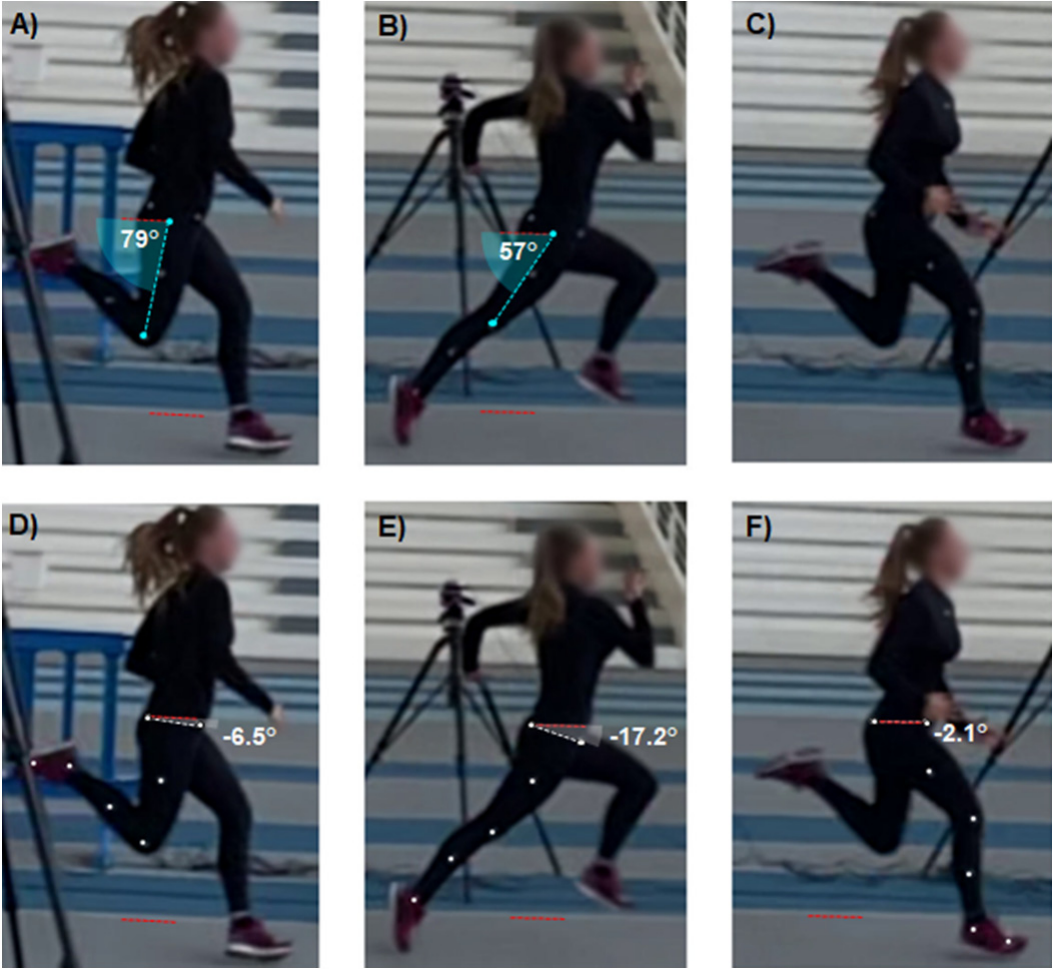
Thigh and pelvic tilt angles were analysed based on sagittal plane images at two specific events of the sprint cycle: toe-off of the ipsilateral leg (A and D), and foot strike of the contralateral leg (B and E). The kick-back score was calculated as A+B, and an equivalent composite score for the pelvic tilt was calculated as D+E (referred to as PEL_TO+FS_ in the main text). The pelvic tilt was also measured in the late swing phase (F).

*Experiment I*. After the warm-up, the requisite anthropometric data were collected for incorporation into the lower-limb conventional gait model (CGM, Kadaba et al., 1990). Subsequently, participants were equipped with 16 retro-reflective markers with a 14-mm diameter, positioned on the pelvis and legs in accordance with the CGM model (Figure 1). For reasons of practicality, markers were placed directly on the participants’ tight clothing. Subsequently, a conventional static calibration trial was conducted.

*Experiment II*. After the warm-up, four custom-made 20 mm in diameter reflective markers were fixed with double-sided adhesive tape on the anterior and posterior superior iliac spines, the greater trochanter, and the medial condyle of the knee.

After the warm-up and specific preparation, participants undertook two maximal sprint acceleration runs from a three-point start position. The kinematics of the runs were recorded in 2-D (sagittal plane video, *both experiments*) and 3-D (infrared camera system, *Experiment I*). A one-minute rest interval was provided between the sprints. The sprint with the shortest time to 30 m was identified using the MySprint app (Romero-Franco et al., 2017), which was then selected for further analyses.

To assess the 2-D sagittal kinematics in both *Experiment I* and *II*, the ‘compass’ tool of a free software program, Kinovea (v0.9.5, created in 2009 under the GPLv2 license) was used. This tool has been demonstrated to be a valid and reliable method to assess joint angles from sagittal video recordings (Puig-Diví et al., 2019). Once the horizontal plane has been defined based on a reference frame, the thigh angles and the pelvic tilt were extracted from the 2-D sagittal video in the sprint cycle. This was done with the athlete situated at the midpoint of the image, thus reducing the impact of image distortion on the measured angles. First, the sagittal angle between the ipsilateral thigh (defined as a straight line between the greater trochanter and the lateral epicondyle of the femur) and the horizontal plane was measured at two specific events: the foot strike of the contralateral leg (Figure 1a) and the toe-off of the ipsilateral leg (Figure 1b). The sum of these two angles was then used to calculate the kick-back score (Lahti et al., 2020). Secondly, the pelvic tilt angle was calculated at the same events as the angle of a straight line connecting the anterior and posterior superior iliac spines relative to the horizontal plane (Figure 1d, 1e). The sum of these two angles was then calculated to create a composite score for the pelvic tilt (PEL_TO+FS_). This composite score represents an alternative to the kick-back score. However, it is calculated based on the pelvic tilt angle in comparison to the thigh angle, which is used in the kick-back score. Furthermore, the pelvic tilt was identified in the late swing phase (the last frame preceding foot strike) of the ipsilateral leg (Figure 1f). The frame in question was not precisely at the point at which the hamstrings were at their longest length within the sprint cycle, but rather a few frames after this point. However, the last frame preceding the foot strike is more readily discernible, and the resulting discrepancies in the outcome measures are anticipated to be inconsequential in comparison to inter-individual variations (Franz et al., 2009). In the absence of a history of injury to the left leg, the ipsilateral leg was consistently defined as the right leg.

In *Experiment I*, 3-D kinematics were calculated using the Plug-In-Gait model, which is the commercial version of the CGM model in Vicon Nexus 2.12 software (Oxford, UK). The CGM is a direct kinematic and hierarchical method. The pelvic tilt was extracted from the pelvic kinematics at the same events within the same sprint cycle as the one where the 2-D analyses were performed. Additionally, the length of the biceps femoris muscle-tendon unit relative to the thigh length was calculated in the late swing phase from the regression equation proposed by Hawkins and Hull (1990), as follows:

*L* = 1.048 + 2.09 ∙ 10^-3^ ∙ 𝛼 − 1.6 ∙ 10^-3^ ∙ 𝛽

where L was the normalized length of the biceps femoris muscle-tendon unit, α was the hip flexion angle, and β was the knee flexion angle. The regression coefficients were derived from a modeling approach that was based on the 3-D locations of the origin and the insertion of the muscle, as defined by cadaveric and radiological assessments. Subsequently, the relationship between the muscle-tendon length and the pelvic tilt angle was established.

### **Statistical Analysis**

Statistical analyses were performed using the free statistical platform Jamovi (https://www.jamovi.org).

*Experiment I*. Pearson’s correlation coefficients with 95% confidence intervals (CI) were calculated unless the data were not normally distributed, as tested by the Shapiro-Wilk W test. Spearman’s rho was calculated for non-normally distributed data. In accordance with the *a priori* assumptions, directional hypotheses were tested, which also increased the statistical power of the study. The following assumptions were tested: 1) there would be a negative correlation between the thigh angle and the pelvic tilt as measured by 3-D motion capture at the toe-off and the foot strike; 2) there would be a negative correlation between the kick-back score and PEL_TO+FS_; 3) there would be a positive correlation between the pelvic tilt measured by 2-D and 3-D methods at all three events; and 4) there would be a positive correlation between the pelvic tilt (as measured with both 2-D and 3-D methods) and the length of the biceps femoris in the late swing phase. In all the cases, the statistical significance level was set at α = 0.05. Bland-Altman analyses with 95% limits of agreement were also conducted to assess the agreement between 2-D video-based estimation of the pelvic tilt and the pelvic tilt extracted from 3-D motion analysis.

*Experiment II*. Given the unequal sample sizes of the groups, Welch’s *t*-tests were conducted to compare the kick-back score and the pelvic tilt of recently injured and non-injured players. As in *Experiment I*, we tested directional hypotheses. The statistical hypotheses were that the pelvic tilt would be higher and the kick-back score would be lower in recently injured players than in non-injured players. Cohen’s *d* standardized effect sizes were calculated. The significance level was set at α = 0.05.

## Results

**Figure 2:**
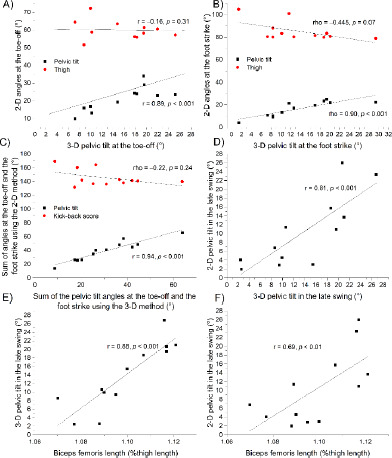
Correlations between 2-D camera-based methods and the pelvic tilt as measured using 3-D motion analysis (A–D), and correlations between the pelvic tilt and biceps femoris muscle-tendon length (E–F).

*Experiment I*. No significant correlation was found between the thigh and the 3-D pelvic tilt angles at the ipsilateral toe-off (r = −0.156 [95%CI = −1.0–0.372], *p* = 0.314, Figure 2a) and the contralateral foot strike (Spearman’s rho = −0.445, *p* = 0.074, Figure 2b). Accordingly, no correlation was found between the kick-back score and the 3-D PEL_TO+FS_ (rho = −0.224, *p* = 0.242, Figure 2c).

A significant correlation was observed between the pelvic tilt as measured based on 2-D images and the pelvic tilt as measured by 3-D motion capture at the ipsilateral toe-off (r = 0.890 [0.704–1.0], *p* < 0.001, Figure 2a), the contralateral foot strike (rho = 0.900, *p* < 0.001, Figure 2b), and the ipsilateral late swing (r = 0.808 [0.517–1.0], *p* < 0.001, Figure 2d). Accordingly, a significant correlation was found between the 2-D PEL_TO+FS_ and the 3-D PEL_TO+FS_ (r = 0.944 [0.84–1.0], *p* < 0.001, Figure 2c).

**Figure 3:**
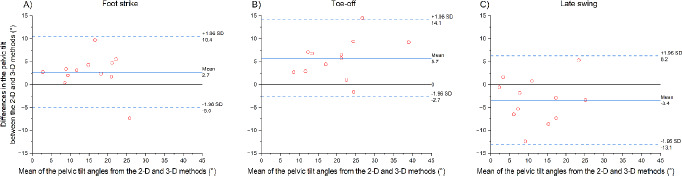
Bland-Altman plots for 2-D sagittal plane camera-based estimation of the pelvic tilt as compared to the pelvic tilt derived from 3-D motion analysis at the foot strike of the contralateral leg (A), the toe-off of the ipsilateral leg (B), and in the late swing phase of the ipsilateral leg (C).

When testing the agreement between the 2-D and 3-D pelvic tilt angles (Figure 3), we found a bias of 2.7 ± 3.9° at the foot strike (limit of agreement, LOA = 7.7°), 5.7 ± 4.3° at the toe-off (LOA = 8.4°), and −3.4 ± 4.9° in the late swing (LOA = 9.6°). In the late swing, there was a significant correlation between the biceps femoris muscle-tendon length and both the 3-D pelvic tilt (r = 0.878 [0.675–1.0], *p* < 0.001, Figure 2e) and the 2-D pelvic tilt (r = 0.685 [0.282–1.0], *p* = 0.007, Figure 2f).

**Figure 4:**
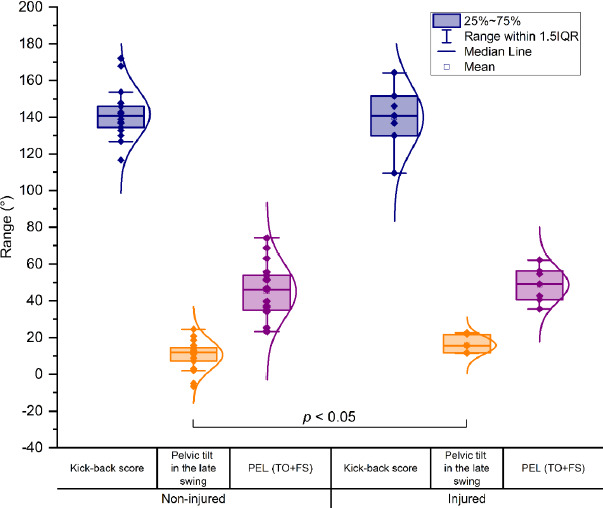
Statistically significant difference in the pelvic tilt was detected between recently injured and non-injured female professional soccer players in the late swing phase of sprinting. However, the composite score for the thigh angle (i.e., kick-back score) and the composite score for the pelvic tilt (PEL_TO+FS_) did not differ significantly between the groups.

*Experiment II*. In the late swing phase, no statistically significant difference was observed in the kick-back score between recently injured and non-injured players (t = 0.241, *p* = 0.407, *d* = 0.114, Figure 4). The anterior pelvic tilt was observed to be higher in the previously injured group than in the non-injured group in the late swing phase (injured = 15.8 ± 4.7 [mean ± standard deviation], non-injured = 10.6 ± 8.1, t = −1.999, *p* = 0.03, *d* = −0.789). On the other hand, no difference was observed between groups in the PEL_TO+FS_ (t = −0.722, *p* = 0.24, *d* = −0.291), as shown in Figure 4.

## Discussion

As anticipated, *Experiment I* revealed a robust correlation between the 2-D video-based approach for the estimation of the pelvic tilt based on a 3-D motion analysis. However, no correlation was observed between the kick-back score and the pelvic tilt. The results also corroborated the hypothesis that there would be a positive correlation between an increased anterior pelvic tilt and an increased biceps femoris muscle-tendon length during the late swing phase of sprinting. *Experiment II* investigated differences in the pelvic tilt in professional female soccer players with and without a recent hamstring injury. The findings revealed a higher anterior pelvic tilt in recently injured players than in non-injured players in the late swing phase of sprinting, as measured by the 2-D camera-based method. On the other hand, no significant difference was found between groups in the kick-back score.

Musculoskeletal modeling has revealed that an increased anterior pelvic tilt increases the length of the hamstrings, which in turn, may increase the risk of hamstring injury (Thelen et al., 2006). In this modeling study, however, the position of the pelvis was modified without alteration to the positions of the thigh or the shank. However, modeling may not reflect what is happening in real-life scenarios. For example, those with an increased anterior pelvic tilt may adjust the position of the thigh and/or minimize knee extension in the late swing phase to protect the hamstrings from excessive lengthening. This speculation does not seem to be justified by the current study, which suggests that an increased pelvic tilt is associated with a longer hamstring (biceps femoris) muscle-tendon length in the late swing, thereby corroborating previous findings based on musculoskeletal modeling. It has recently been shown that following a fatiguing exercise, there is an increase in the angle of the anterior pelvic tilt despite no increase in hamstring muscle-tendon lengths in the late swing (Vial, 2023). This is due to a simultaneous decrease in the hip flexion angle and no change in the angle of the knee (Vial, 2023). It can be inferred that the level of fatigue should be controlled when assessing the pelvic tilt as a risk factor for hamstring injuries.

In previous studies, considerable variability in the pelvic tilt angle has been documented, with values ranging from −3° to 35°, depending on the running event, sprint distance, and running technique (Mendiguchia et al., 2022; Nagahara et al., 2018; Schuermans et al., 2017b). Despite considerable inter-athlete variability, the mean values observed in the current study (*Experiment I*, pelvic tilt: at the toe-off = 13.8°, foot strike = 13.7°, late swing = 13.8°) were similar to those reported by Schuermans et al. (2017b; 16° at the toe-off and 14° in the late swing) in non-injured soccer players. These comparisons are based on 3-D motion capture used in both studies.

As an alternative to the conventional 3-D motion capture setup, the measurement of the kick-back score based on a single video camera recording has been introduced (Lahti et al., 2020). It has been assumed that there is a correlation between the position of the thigh and the position of the pelvis at discrete points in the sprint cycle. In their study, Lahti et al. (2020) reported a kick-back score of approximately 146° and 143°, in non-injured and injured male soccer players, respectively, with no significant difference between groups. These values are in close proximity to those observed in the current study (*Experiment I*: 145° group mean; *Experiment II*: 142° in non-injured and 140° in recently injured players). Based on the results of the current study, there is no evidence to suggest a correlation between the pelvic tilt and thigh angles (i.e., the kick-back score). It seems that a given thigh angle can manifest along with a range of pelvic tilt angles. For example, participants #9 and #10 exhibited a comparable kick-back score of 142° and 140°, respectively, yet displayed notable discrepancies in the 3-D pelvic tilt at the toe-off (10.2° vs. 34.4°), the foot strike (10.2° vs. 29.4°), and the late swing (9.4° vs. 26.8°). It is therefore proposed that the kick-back score should not be used as a measure of the pelvic tilt.

To address the constraints of 3-D motion capture, a considerable body of research has been conducted to ascertain the reliability of video-based techniques for the evaluation of lower limb kinematics in the sagittal plane. These were tested across a spectrum of motor activities, including a single-leg squat (Schurr et al., 2017), walking (Ugbolue et al., 2013), and running (Peebles et al., 2021). To the best of our knowledge, no studies have been conducted to validate the measurement of the pelvic tilt during sprinting. We found a correlation between 2-D video-based and 3-D motion analysis methods. It should be noted that a bias was identified at each sprint event (Figure 3). These appear to be comparable to those reported in previous studies that assessed the concurrent validity of inertial measurement units (2.8° root-mean-square error; Wada et al., 2020) or markerless methods (3.8° root-mean-square error; Castelli et al., 2015) during sprinting or fast walking. The notable bias may be attributed to the failure to incorporate pelvic rotation and obliquity into the 2-D method. Additional experimental analysis from 3-D motion capture revealed that the rotation and the obliquity were considerable at the foot strike (6.7° and −5.2°, respectively), the toe-off (−6.2° and −8.9°, respectively), and the late swing (−7.1° and 5.9°, respectively). These observations are consistent with those reported in previous studies (Ota et al., 2021; Sado et al., 2017). Nevertheless, the high correlations indicate that the 2-D video-based method with two markers on the pelvis is a reliable alternative to the 3-D motion capture for assessing inter-individual differences or monitoring the athletes’ pelvic tilt during sprinting throughout a season.

The 2-D marker-based method revealed that previously injured female soccer players exhibited a greater degree of the anterior pelvic tilt in the late swing phase than their non-injured counterparts. However, the kick-back score was found to be comparable between these two groups (Figure 4). These results are comparable to those previously reported in male soccer players, despite the existence of anatomical differences between the sexes. It is noteworthy that there was no significant difference between groups in the PEL_TO+FS_, which score was derived from the pelvic tilt at events where the kick-back score was calculated. This may be due to the fact that the toe-off is not a typical event for hamstring injuries, and the contralateral leg is still in the mid-swing at this moment, which is also a phase that is unrelated to hamstring injuries. In these phases the knee joint is relatively flexed, thereby reducing the potential adverse effects of an increased pelvic tilt on the length of the hamstrings. On the contrary, in the late swing phase, the knee is relatively more extended. Therefore, the assessment of the pelvic tilt should prioritize the late swing phase over the early swing phase or other phases of sprinting in the context of hamstring injury management.

As a limitation of the study, the markers were placed on tight-fitting clothing rather than directly on the skin, which may have introduced additional artifacts. Nevertheless, our objective was to implement a configuration that could be readily operationalized in field settings. It is also noteworthy that the kinematic data were recorded at a distance of 15 m from the sprint start, which may have been insufficient for some athletes to achieve their maximum running speed. Nevertheless, we assume that the effect was relatively minor in both studies given that the pelvic tilt during late acceleration appears to be comparable to the pelvic tilt at maximum speed running (Sado et al., 2017). It is also important to note that biceps femoris length estimation does not account for potential anatomical variations between individuals, and it refers to the entire muscle-tendon unit. However, additional factors, including muscle-tendon decoupling, fascicle gearing, and regional differences within the muscle, also influence muscle fibre length. It is therefore imperative that any direct conclusions drawn from pelvic mechanics to fascicle or fibre mechanics should be approached with a high degree of caution. The relatively small sample size in these experiments may have resulted in the increased likelihood of type II errors and overrepresentation of the effects or differences when a statistically significant result was observed. Consequently, the findings of this study should be validated by future research using larger sample sizes. Such a design would also facilitate for the control of confounding variables when comparing athletes with and without a history of injury.

## Conclusions

Our findings, derived from experimental analysis, corroborate the notion that an excessive anterior pelvic tilt is associated with an increased biceps femoris length in the late swig phase of sprinting. This lends support to the notion that monitoring pelvic tilt in sprint-based sports is crucial from an injury prevention perspective not only in male athletes (as previously suggested), but also in female athletes. It would be beneficial to investigate whether a reduction in the anterior pelvic tilt in the late swing phase of sprinting during the rehabilitation process could potentially prevent hamstring re-injuries. In field settings, there is a need for straightforward methods. However, some approaches such as the kick-back score may be overly simplistic. Conversely, the pelvic tilt can be monitored using a single video camera recording in the sagittal plane based on markers on the anterior and posterior superior iliac spines. Despite some bias, the correlations were robust, and this method enabled the detection of differences between recently injured and non-injured female professional soccer players when the analysis focused on the late swing phase of the sprint. Further prospective studies on female players monitoring the pelvic tilt are required, and the 2-D approach introduced in the current study can streamline the relatively complex setup of 3-D motion capture to measure the pelvic tilt angle.

## References

[ref1] Blackburn, J. T., Bell, D. R., Norcross, M. F., Hudson, J. D., & Kimsey, M. H. (2009). Sex comparison of hamstring structural and material properties. Clinical Biomechanics, 24(1), 65–70. 10.1016/j.clinbiomech.2008.10.001 19026473

[ref2] Bourne, M. N., Opar, D. A., Williams, M. D., & Shield, A. J. (2015). Eccentric Knee Flexor Strength and Risk of Hamstring Injuries in Rugby Union: A Prospective Study. American Journal of Sports Medicine, 43(11), 2663–2670. 10.1177/0363546515599633 26337245

[ref3] Castelli, A., Paolini, G., Cereatti, A., & Della Croce, U. (2015). A 2D Markerless Gait Analysis Methodology: Validation on Healthy Subjects. *Computational and Mathematical Methods in Medicine*, 2015, e186780. 10.1155/2015/186780 PMC4430646 26064181

[ref4] Daly, C., McCarthy Persson, U., Twycross-Lewis, R., Woledge, R. C., & Morrissey, D. (2016). The biomechanics of running in athletes with previous hamstring injury: A case-control study. Scandinavian Journal of Medicine & Science in Sports, 26(4), 413–420. 10.1111/sms.12464 25913546

[ref5] Fischer, B. & Mitteroecker, P. (2017). Allometry and Sexual Dimorphism in the Human Pelvis. Anatomical Record, 300(4), 698–705. 10.1002/ar.23549 28297185

[ref6] Franz, J. R., Paylo, K. W., Dicharry, J., Riley, P. O., & Kerrigan, D. C. (2009). Changes in the coordination of hip and pelvis kinematics with mode of locomotion. Gait & Posture, 29(3), 494–498. 10.1016/j.gaitpost.2008.11.011 19124245

[ref7] Garrett, W. E., Safran, M. R., Seaber, A. V., Glisson, R. R., & Ribbeck, B. M. (1987). Biomechanical comparison of stimulated and nonstimulated skeletal muscle pulled to failure. American Journal of Sports Medicine, 15(5), 448–454. 10.1177/036354658701500504 3674268

[ref8] Hawkins, D., & Hull, M. L. (1990). A method for determining lower extremity muscle-tendon lengths during flexion/extension movements. Journal of Biomechanics, 23(5), 487–494. 10.1016/0021-9290(90)90304-l 2373721

[ref9] Higashihara, A., Nagano, Y., Takahashi, K., & Fukubayashi, T. (2015). Effects of forward trunk lean on hamstring muscle kinematics during sprinting. Journal of Sports Sciences, 33(13), 1366–1375. 10.1080/02640414.2014.990483 25514378

[ref10] Higashihara, A., Ono, T., Tokutake, G., Kuramochi, R., Kunita, Y., Nagano, Y., & Hirose, N. (2019). Hamstring muscles’ function deficit during overground sprinting in track and field athletes with a history of strain injury. Journal of Sports Sciences, 37(23), 2744–2750. 10.1080/02640414.2019.1664030 31608831

[ref11] Horan, D., Blake, C., Hägglund, M., Kelly, S., Roe, M., & Delahunt, E. (2022). Injuries in elite-level women’s football—A two-year prospective study in the Irish Women’s National League. Scandinavian Journal of Medicine & Science in Sports, 32(1), 177–190. 10.1111/sms.14062 34719066

[ref12] Kadaba, M. P., Ramakrishnan, H. K., & Wootten, M. E. (1990). Measurement of lower extremity kinematics during level walking. Journal of Orthopaedic Research, 8(3), 383–392. 10.1002/jor.1100080310 2324857

[ref13] Kenneally-Dabrowski, C., Brown, N. A., Warnenhoven, J., Serpell, B. G., Perriman, D., Lai, A. K. M., Spratford, W. (2019). Late swing running mechanics influence hamstring injury susceptibility in elite rugby athletes: a prospective exploratory analysis. Journal of Biomechanics, 92, 112–119. 31176462 10.1016/j.jbiomech.2019.05.037

[ref14] Lahti, J., Mendiguchia, J., Ahtiainen, J., Anula, L., Kononen, T., Kujala, M., Matinlauri, A., Peltonen, V., Thibault, M., Toivonen, R.-M., Edouard, P., & Morin, J. B. (2020). Multifactorial individualised programme for hamstring muscle injury risk reduction in professional football: Protocol for a prospective cohort study. *BMJ Open Sport & Exercise Medicine*, 6(1), e000758. 10.1136/bmjsem-2020-000758 PMC10860081 38347859

[ref15] Lieber, R. L. and Fridén, J. (1993). Muscle damage is not a function of muscle force but active muscle strain. Journal of Applied Physiology (Bethesda, Md.: 1985), 74(2), 520–526. 10.1152/jappl.1993.74.2.520 8458765

[ref16] Mendiguchia, J., Castaño-Zambudio, A., Jiménez-Reyes, P., Morin, J.-B., Edouard, P., Conceição, F., Tawiah-Dodoo, J., & Colyer, S. L. (2022). Can We Modify Maximal Speed Running Posture? Implications for Performance and Hamstring Injury Management. International Journal of Sports Physiology and Performance, 17(3), 374–383. 10.1123/ijspp.2021-0107 34794121

[ref17] Nagahara, R., Mizutani, M., Matsuo, A., Kanehisa, H., & Fukunaga, T. (2018). Step-to-step spatiotemporal variables and ground reaction forces of intra-individual fastest sprinting in a single session. Journal of Sports Sciences, 36(12), 1392–1401. 10.1080/02640414.2017.1389101 28988513

[ref18] Nagano, Y., Higashihara, A., Takahashi, K., & Fukubayashi, T. (2014). Mechanics of the muscles crossing the hip joint during sprint running. Journal of Sports Sciences, 32(18), 1722–1728. 10.1080/02640414.2014.915423 24840031

[ref19] Nilstad, A., Andersen, T. E., Bahr, R., Holme, I., & Steffen, K. (2014). Risk factors for lower extremity injuries in elite female soccer players. American Journal of Sports Medicine, 42(4), 940–948. 10.1177/0363546513518741 24500914

[ref20] Ota, M., Tateuchi, H., Hashiguchi, T., & Ichihashi, N. (2021). Verification of validity of gait analysis systems during treadmill walking and running using human pose tracking algorithm. Gait & Posture, 85, 290–297. 10.1016/j.gaitpost.2021.02.006 33636458

[ref21] Peebles, A. T., Arena, S. L., & Queen, R. M. (2021). A new method for assessing landing kinematics in non-laboratory settings. Physical Therapy in Sport, 49, 21–30. 10.1016/j.ptsp.2021.01.012 33550202

[ref22] Puig-Diví, A., Escalona-Marfil, C., Padullés-Riu, J. M., Busquets, A., Padullés-Chando, X., & Marcos-Ruiz, D. (2019). Validity and reliability of the Kinovea program in obtaining angles and distances using coordinates in 4 perspectives. *PloS One*, 14(6), e0216448. 10.1371/journal.pone.0216448 PMC6550386 31166989

[ref23] Romero-Franco, N., Jiménez-Reyes, P., Castaño-Zambudio, A., Capelo-Ramírez, F., Rodríguez-Juan, J. J., González-Hernández, J., Toscano-Bendala, F. J., Cuadrado-Peñafiel, V., & Balsalobre-Fernández, C. (2017). Sprint performance and mechanical outputs computed with an iPhone app: Comparison with existing reference methods. European Journal of Sport Science, 17(4), 386–392. 10.1080/17461391.2016.1249031 27806673

[ref24] Sado, N., Yoshioka, S., & Fukashiro, S. (2017). The three-dimensional kinetic behaviour of the pelvic rotation in maximal sprint running. Sports Biomechanics, 16(2), 258–271. 10.1080/14763141.2016.1231837 27846785

[ref25] Schuermans, J., Danneels, L., Van Tiggelen, D., Palmans, T., & Witvrouw, E. (2017a). Proximal Neuromuscular Control Protects Against Hamstring Injuries in Male Soccer Players: A Prospective Study With Electromyography Time-Series Analysis During Maximal Sprinting. American Journal of Sports Medicine, 45(6), 1315–1325. 10.1177/0363546516687750 28263670

[ref26] Schuermans, J., Van Tiggelen, D., Palmans, T., Danneels, L., & Witvrouw, E. (2017b). Deviating running kinematics and hamstring injury susceptibility in male soccer players: Cause or consequence? Gait & Posture, 57, 270–277. 10.1016/j.gaitpost.2017.06.268 28683419

[ref27] Schurr, S. A., Marshall, A. N., Resch, J. E., & Saliba, S. A. (2017). Two-dimensonal video analysis is comparable to 3D motion capture in lower extremity movement assessment. International Journal of Sports Physical Therapy, 12(2), 163–172. 28515970 PMC5380858

[ref28] Thelen, D. G., Chumanov, E. S., Sherry, M. A., & Heiderscheit, B. C. (2006). Neuromusculoskeletal Models Provide Insights into the Mechanisms and Rehabilitation of Hamstring Strains. *Exercise and Sport Sciences Reviews*, 34(3), 135. 16829741 10.1249/00003677-200607000-00008

[ref29] Timmins, R. G., Bourne, M. N., Shield, A. J., Williams, M. D., Lorenzen, C., & Opar, D. A. (2016). Short biceps femoris fascicles and eccentric knee flexor weakness increase the risk of hamstring injury in elite football (soccer): A prospective cohort study. British Journal of Sports Medicine, 50(24), 1524–1535. 10.1136/bjsports-2015-095362 26675089

[ref30] Ugbolue, U. C., Papi, E., Kaliarntas, K. T., Kerr, A., Earl, L., Pomeroy, V. M., & Rowe, P. J. (2013). The evaluation of an inexpensive, 2D, video based gait assessment system for clinical use. Gait & Posture, 38(3), 483–489. 10.1016/j.gaitpost.2013.01.018 23465758

[ref31] Vial, S. (2023). The effect of performance fatigue on sprint running technique. *Theses: Doctorates and Masters*. https://ro.ecu.edu.au/theses/2626; accessed on 29 November 2023

[ref32] Wada, T., Nagahara, R., Gleadhill, S., Ishizuka, T., Ohnuma, H., & Ohgi, Y. (2020). Measurement of Pelvic Orientation Angles during Sprinting Using a Single Inertial Sensor. *Proceedings*, 49(1), 1. 10.3390/proceedings2020049010

[ref33] Watsford, M. L., Murphy, A. J., McLachlan, K. A., Bryant, A. L., Cameron, M. L., Crossley, K. M., & Makdissi, M. (2010). A prospective study of the relationship between lower body stiffness and hamstring injury in professional Australian rules footballers. American Journal of Sports Medicine, 38(10), 2058–2064. 10.1177/0363546510370197 20595555

